# Relation Between Familial Mediterranean Fever and QT Markers (QTc, QTd, and QTcd): A Systematic Review and Meta-Analysis

**DOI:** 10.7759/cureus.30585

**Published:** 2022-10-22

**Authors:** Karam R Motawea, Amro A El-Sakka, Omneya A Kandil, Nancy Ahmed, Merna Abdelnaem, Bishoy Zaki, Rowan H Elhalag, Joseph Varney, Sarya Swed, Abdulqadir J Nashwan, Bisher Sawaf, Mohamed Seijari, Amr Farwati, Naim Battikh, Amine Rakab

**Affiliations:** 1 Medicine, Alexandria University, Alexandria, EGY; 2 Medicine, Suez Canal University, Ismailia, EGY; 3 Medicine, Ain Shams University, Cairo, EGY; 4 Medicine, American University of the Caribbean, School of Medicine, New York City, USA; 5 Medicine, Aleppo University, Aleppo, SYR; 6 Nursing, Hamad Medical Corporation, Doha, QAT; 7 Internal Medicine, Faculty of Medicine, Syrian Private Unviersity, Damascus, SYR; 8 Medical Education, Hamad Medical Corporation, Doha, QAT; 9 Internal Medicine, Hamad Medical Corporation, Doha, QAT; 10 Medicine, Jr. Hospital of Cook County, Chicago, USA; 11 Internal Medicine, Weill Cornell Medicine - Qatar, Education City, QAT

**Keywords:** meta-analysis, systematic review, familial mediterranean fever, arrhythmia, qt markers

## Abstract

The aim of this study is to perform a meta-analysis to evaluate the possible association betweenQT markers and familial Mediterranean fever (FMF). PUBMED, Web of Science, OVID, and SCOPUS databases were searched. Inclusion criteria were randomized control trials or observational studies that compared measurement of the QT markers in FMF patients and healthy controls in both males and females without any age restriction or other comorbidities. RevMan software (5.4) was used to perform the analysis. A total of 14 studies with 1,154 individuals were included in the study. The pooled effect estimate showed a statistically significant association between FMF group and prolonged corrected QT (QTc) and QT dispersion (QTd) (MD= 7.06, 95% CI = 2.68 to 11.43, p-value = 0.002) and (MD= 6.08, 95% CI = 0.84 to 11.32, p-value= 0.02), respectively. No statistically significant difference between FMF group and QT interval and corrected QT dispersion (QTcd) (MD= 2.34, 95% CI = -1.21 to 5.89, p-value = 0.20) and (MD= 4.82, 95% CI = -0.57 to 10.20, p-value = 0.08), respectively. Our findings revealed a statistically significant relationship between FMF and extended QTc and QTd. More randomized multicenter trials are required to confirm our findings.

## Introduction and background

Familial Mediterranean fever (FMF) is an autosomal recessive auto-inflammatory disease featuring recurrent bouts of fever, abdominal pain, and arthritis along with serous membrane inflammation, which varies in duration from hours to days and occurs at variable intervals [1]. FMF is diagnosed using Tel-Hashomer clinical criteria, which should compromise two or more major symptoms, including (febrile episodes with serositis or a favorable response to colchicine or amyloidosis), range and pattern of fever: recurrent (at least three episodes), febrile (rectal temperature ≥ 38 °C) and short in duration (12 hours to three days). Incomplete attacks (must be recurrent) are defined as differing from typical attacks in one or two features as follows: 1) temperature <38 °C, 2) attack duration longer or shorter than a typical attack (but no less than six hours and no more than seven days), or one major plus two minor symptoms, including (a first-degree relative with FMF, erysipelas like erythema and recurrent febrile episodes). FMF is more prevalent in Turks, Armenians, Italians, Mediterranean, and Middle Eastern descent than in Greeks, Iranians, and Jews. For instance, its prevalence in Turkey is estimated to be one in 1,000 children. Mediterranean fever (MEFV) gene [1-3] mutation is thought to be responsible for its occurrence.

The constant inflammatory process - even in between attacks - has a destructive effect on the heart and may disrupt the course of ventricular repolarization, leading to increased dispersion of recovery time throughout the ventricle (high QT dispersion [QTd]), which also happens in other inflammatory diseases such as SLE. Additionally, amyloidosis due to FMF can be considered a confounding factor for arrhythmia development.

Several studies have specifically investigated repolarization and markers changes in FMF patients. Available markers are the QT interval representing the time from the beginning of depolarization till the end of repolarization in the chosen lead axis. QTc is calculated using the Bazzett formula and developed to yield a corrected measure since the QT interval varies with heart rate. QTd is the difference between the longest (QT max) and the shortest QT, while corrected QT dispersion (QTcd) is its counterpart but corrected for heart rate. Repolarization abnormalities marked by a high QTd, or QT variability are arrhythmogenic, as they increase the risk of developing a tachyarrhythmia called Torsades de Pointes which may lead to ventricular fibrillation and sudden cardiac death [4-6].

Contradictory results were reported regarding these markers in FMF. Studies have contrasting inferences on the value of QT markers in FMF; some studies claimed no difference between FMF patients and controls [7,8], while others found high QTd in these patients [9]. Moreover, some studies reported a higher QTc value in FMF patients than in controls [10]. The aim of this systematic review and meta-analysis is to pool the available data to find if there is an actual association between FMF and abnormal QT markers.

This article was previously presented as a meeting abstract at the 6th Heart in Diabetes Conference on June 24, 2022, in Philadelphia, PA, USA.

## Review

Methods

Search Strategy

We searched the following databases: PUBMED, Web of Science, OVID, and SCOPUS. The terms used during the search were (“Familial Mediterranean Fever” OR “Familial Paroxysmal Polyserositis” OR “Mediterranean Fever, Familial” OR “Periodic Disease” OR “Periodic Peritonitis” OR “Recurrent Polyserositis” OR “Benign Paroxysmal Peritonitis”) AND (“Torsades de Pointes” OR “QT interval” OR “QT”).

Definition of Variables

QTd is the difference between the maximum QT value and the minimum QT value measured with a 12-derivation surface electrocardiogram (ECG). It has been proposed as a non-invasive ECG parameter for inspecting the homogeneity of ventricular recovery time. Since increased QTd shows heterogeneity in ventricular repolarization, it is conclusively associated with increased liability for developing ventricular arrhythmia and sudden cardiac death. Additionally, Corrected QT (QTc) represents ventricular repolarization and is measured as the difference between the longest and shortest QTc interval on surface electrocardiography (ECG). It refers to the QT interval adjusted for the heart rate. QTc can forecast the risk of developing malignant arrhythmias as its prolongation may lead to fatal ventricular arrhythmias. On the other hand, QTcd is calculated as the difference between the maximum and the minimum QTc distances in milliseconds by any ECG derivation.

Inclusion Criteria and Selection Process 

Yielded results from databases were imported into the Covidence platform. We reviewed the title and abstract of each paper from the searches and retrieved potentially relevant references. Following this initial screening, we obtained the full text of potentially relevant studies. The full-text screening was done for the papers using predetermined inclusion criteria which are any randomized control trials or observational studies that compared measurement of the QT markers (QTc, QTd, and QTcd) in FMF patients and healthy controls in both males and females without any age restriction or any other comorbidities. Meanwhile, reviews, case reports, editorials, and animal studies were excluded. Any conflicts about study inclusions were resolved by consensus.

Data Extraction and Quality Assessment 

Two authors (MA and NI) independently extracted data and consulted the first author (KRM) when needed. They extracted details of the study design, participant characteristics, intervention and comparator, and outcomes. Quality assessment was done by the New Castle Ottawa scale assessment tool. Studies quality was ranked as good, fair, or poor.

Statistical Analysis

A meta-analysis was performed to assess the relationship between FMF and QT anomalies. For statistical analysis, RevMan 5.4 was employed. To ensure our analysis's high quality, we used the PRISMA Statement checklist. The continuous outcomes were quantified with a 95% confidence interval as mean difference (MD) and standard deviation (SD). If the P-value was less than 0.05, the findings were deemed significant.

Clinical Criteria of FMF

FMF is diagnosed by Tel-Hashomer clinical criteria, which should compromise two or more major symptoms, including (febrile episodes with serositis or a favorable response to colchicine or amyloidosis) or one major plus two minor symptoms, including (a first-degree relative with FMF, erysipelas like erythema and recurrent febrile episodes).

Results

Literature Search

**Figure 1:**
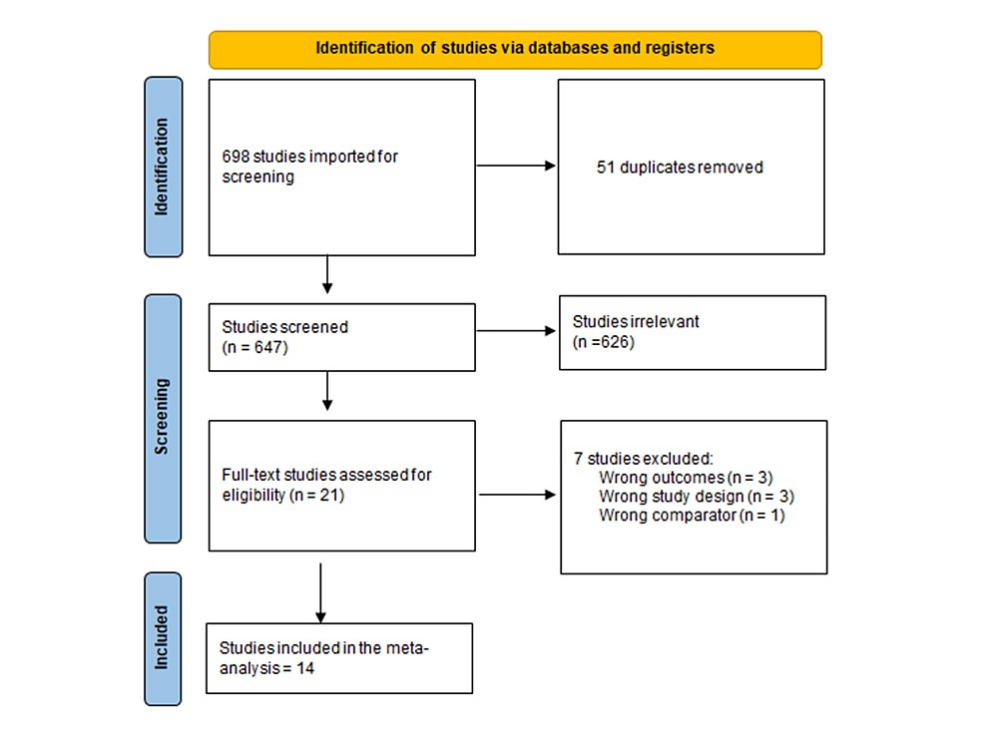
PRISMA flow diagram

After completing the literature search, 698 publications resulted, and then 647 were deemed eligible for the title and abstract screening after removing duplicates. Of the 647, 21 were eligible for full-text screening. After the full-text screening, 14 studies were included in the meta-analysis (7-10,14-18, 27-31), and seven were excluded (32-38) for different reasons, as shown in Figure 1.

Characteristics

QT, QTc, QTd, and QTcd outcomes were reported in 9, 13, 12, and 11 studies, respectively. Most of the studies were of good quality (11 studies), one study was of fair quality, and only two had poor quality. The quality assessment summary is shown in Table 1.

**Table 1:** NOS scale risk of bias assessment NOS: Newcastle-Ottawa Scale; AHRQ: Agency for Healthcare Research and Quality

	Selection				Comparability	Exposure			Total Score	AHRQ Standards
Study	Case definition	Representativeness	Selection of controls	Definition of controls		Ascertainment	Same method	Non-response rate		
1) Nussinovitch, 2011	1	0	1	1	2	1	1	1	8	good
2) Fidanci, 2015	1	1	1	1	2	1	1	1	9	good
3) Nussinovitch, 2012	1	0	1	1	2	1	0	1	7	good
4) Koca, 2011	1	1	1	1	2	1	0	1	8	good
5) Topal, 2011	1	1	1	1	2	1	1	1	9	good
6) Sahin, 2014	1	1	1	1	0	1	0	1	6	poor
7) Akcay, 2009	1	1	1	1	0	1	0	1	6	poor
8) Nussinovitch, 2012	1	0	1	1	2	1	0	1	7	good
9) Nussinovitch, 2010	1	0	0	1	2	1	0	1	6	fair
10) Kirbas, 2016	1	1	1	1	2	1	0	1	8	good
11) Canpolat, 2011	1	1	1	1	2	1	1	1	9	good
12) Karaman, 2017	1	1	0	1	2	1	0	1	7	good
13) Giese, 2014	1	1	1	1	2	1	1	1	9	good
14) Ahbap, 2015	1	1	1	1	2	1	0	1	8	good

The total number of patients included in the meta-analysis is 1154,603 in the FMF group and 551 in the control group; other baseline characteristics are found in Table 2.

**Table 2:** Baseline characteristics of the eligible studies

Study	Duration of the disease " mean, SD"	Study arms	Endpoints (outcomes)	Conclusion
1) Nussinovitch, 2011	_	The study group consisted of 18 patients (7 females) with FMF diagnosed according to the diagnostic criteria. Eighteen healthy volunteers matched for sex and age served as controls.	There were no statistically significant differences between the groups as to average corrected QT interval length, average QTd interval, average QT corrected dispersion or QT dispersion ratio. JT dispersion and JT corrected dispersion were also similar in both groups.	The patients with FMF- amyloidosis seem to have QT and JT dispersion parameters similar to those of healthy subjects. Future research and longer follow-up should be conducted in order to evaluate the prognostic importance of repolarization dispersion parameters in amyloidosis of FMF.
2) Fidanci, 2015	48 (5.2) "months"	The study included 48 FMF patients as FMF patients and 31 healthy children as the healthy controls.	There was no statistically significant difference between the FMF patients and healthy controls in terms of RR, QT, QTd, QTcd, JT, JTc, JTd, and JTcd measurements and echocardiography parameters. QTc value was higher in the FMF patients than in the healthy controls.	QTc value indicates increased ventricular sensitivity and is an important marker of cardiovascular mortality. It has an important effect on sudden cardiac death and arrhythmia. Our study results suggest that electrocardiographic monitoring may be useful in patients with FMF.
3) Nussinovitch, 2012	_	The study group included 53 patients diagnosed with FMF and recruited from the outpatient clinic. Fifty-three healthy subjects, matched for sex and age, served as controls. None of the patients or controls was taking medications known to influence QT interval duration or depolarization.	No significant difference in any of the QT dynamic parameters was found in either FMF group compared with the healthy controls. Mean values of the QT variability index, regardless of colchicine response, were similar to previously published results for healthy persons.	The patients with FMF who are continuously treated with colchicine and have not developed amyloidosis, regardless of their clinical response, have normal QT variability parameters, indicating normal repolarization dynamics and suggesting no increased risk of repolarization-associated cardiac arrhythmias.
4) Koca, 2011	5.6 (2.8) "years"	Participants included children and adolescents (6 to 17 years old). Thirty- five patients (17 females and 18 males) with FMF and 35 healthy controls (15 females and 20 males) were enrolled in the study.	The heart rate recovery (HRR) indices of the two groups were similar. Also, the chronotropic response was similar in both groups. The time-domain parameters of heart rate variability (HRV) were similar in both groups, except for mean RR (p=0.024). Frequencies of ventricular and supraventricular ectopic stimuli were similar in both groups. There were no statistically significant differences between the groups in average QT and average corrected QT interval length, average QT interval dispersion, and average QT corrected dispersion. There was no significant difference between the two groups regarding the ratio of clinical dysautonomic reactions on HUTT. However, we observed a significantly higher rate of dysautonomic reactions on HUTT in patients with exertional leg pain than that in patients without (p=0.013). When the fractal dimension of time curves was compared, FMF patients exhibited significantly lower diastolic blood pressure parameters than controls in response to HUTT.	This study suggested that cardiovascular autonomic dysfunction in children with FMF is not prominent as in adult patients. Particularly, patients with exertional leg pain are more prone to have dysautonomic features.
5) Topal, 2011	_	Thirty-eight FMF patients and 35 healthy control subjects were recruited to the study.	There were no significant between-group differences in any other measured variable. The median duration of both disease and drug treatment was 6 years (range 1 – 35 years). Proteinuria was present in 10.3% of FMF patients, but none had a renal failure or biopsy-proven amyloidosis. Inter- and intraobserver variability in QT measurement was < 5%. The ventricular repolarization parameters (QT interval and QT dispersion) are given in Table 2. There were no statistically significant between-group differences in any ventricular repolarization parameter.	This study demonstrated that electrocardiographic ventricular repolarization parameters (QT interval and QT dispersion) are of little value for the evaluation of cardiac impairment and risk of arrhythmia in FMF patients.
6) Sahin, 2014	78.6 (85.5) "months"	A total of 50 FMF patients in an attack-free period (30 men, 20 women; mean age 29.4 ± 11.8 years) according to the Tel-Hashomer criteria were selected from the rheumatology outpatient clinic of our university hospital. Fifty healthy control subjects (30 men, 20 women; mean age31.3 ± 11.9 years) were recruited from our cardiology outpatient clinics.	The QTd, QTmax, and TDR were greater in FMF patients than in the control group (36.0 ± 11.4 vs. 20 ± 11.2, P\0.001 and 354.8 ± 30.9vs.342.8 ± 18.0, P = 0.02;62.0 ± 16.0vs.49.0 ± 9.5 P\0.001, respectively), as were cQTd and cQTmax (40.4 ± 13.5 vs. 21.9 ± 12.4, P\0.001 and 397.7 ± 40.2 vs. 375.5 ± 25.4 P = 0.001). A modest positive correlation was found between cQTd and C-reactive protein (CRP) and erythrocyte sedimentation rate (ESR)(r = 0.30, P\0.001; r = 0.40, P\0.001; respectively). QTd, which is an index of inhomogeneity of ventricular repolarization and an important predictor of cardiovascular mortality, and TDR, which is a better marker of cardiac repolarization, increased in FMF patients similarly as in other rheumatologic diseases	QTd, which is an index of homogeneity of ventricular repolarization and an important predictor of cardiovascular mortality, and TDR, which is a better marker of cardiac repolarization, increased in FMF patients similarly as in other rheumatologic diseases.
7) Akcay, 2009	_	Twenty-two FMF patients and 22 age-and sex-matched control subjects were included in the study.	Both FMF patients and controls had similar comorbidities, similar values of average QT, average corrected QT interval length, average QTd interval, average QT corrected dispersion, QT dispersion ratio, JT dispersion (JTd), and JT corrected dispersion.	FMF patients who were unresponsive to colchicine treatment and did not develop amyloidosis had normal QTd and JTd parameters, indicating a non-increased risk for repolarization-associated ventricular arrhythmias.
8) Nussinovitch, 2012	_	The study group consisted of32 patients (18 females) with FMF diagnosed according to the TelHashomer criteria. Thirty-seven healthy subjects matched for sex and age served as the control group.	There were no statistically significant differences between the groups in average QT and average corrected QT interval length, average QT interval dispersion, average QT corrected dispersion or QT dispersion ratio. During 6 months of follow-up, no cases of sudden death or arrhythmia were documented in either group. Patients with FMF who are continuously treated with low-dose colchicine and have not developed amyloidosis seem to have QT dispersion parameters similar to those of healthy subjects and, therefore, apparently, have no increased risk of adverse cardiac events associated with abnormal repolarization.	Patients with FMF who are continuously treated with low-dose colchicine and have not developed amyloidosis appear to have similar QT dispersion parameters to those of healthy controls. In light of the disagreement between our results and those of an earlier study, we suggest further research to evaluate the association between early detection of atherosclerosis, the genetic basis for FMF phenotype, electrocardiographic markers, and long-term cardiovascular risk and complications.
9) Nussinovitch, 2010	5.2 (2.4) "years"	In this case-control study, 37 pregnant women with FMF who had already been put on colchicine treatment and 40 healthy, uncomplicated pregnancy cases were prospectively assessed using 12-lead ECG and echocardiography.	No differences in Pd and corrected QT values were found between the groups. Epicardial fat thickness values were significantly higher in the FMF group compared with the control group (p = 0.015). A positive correlation was found between FMF duration and epicardial fat thickness (r = 0.350, p = 0.042).	Pd, a noninvasive marker of potential atrial arrhythmia, and QT-d, a noninvasive marker of potentially lethal ventricular tachyarrhythmia, constitute a recent contribution to the field of noninvasive electro-cardiology. Pd and QT-d values were not altered in pregnant women with FMF who had already been put on colchicine treatment, with no increased risk of atrial or ventricular arrhythmias indicated. Colchicine may have a cardio-protective effect beyond the effect mediated through the suppression of inflammation.
10) Kirbas, 2016	9.8 (4.2) "years"	The study included 38 patients with FMF and 34 healthy subjects as controls.	Both groups were similar with regard to baseline characteristics. Mean HRR1 (p= 0.001), HRR2 (p= 0.003) and HRR3 (pb0.001) were significantly lower in FMF group. SDNN (standard deviation of all NN intervals), SDANN (SD of the 5 min mean RR intervals), RMSSD (root square of successive differences in RR interval), and PNN50 (proportion of differences in successive NN intervals >50 ms) and high frequency (HF) components were significantly decreased, but the low frequency (LF) and LF/HF were significantly higher in FMF patients. HRT onset and slope were significantly less negative in FMF patients. Also, QTd was significantly higher in FMF patients (pb0.001).	Patients with FMF showed delayed recovery of heart rate and abnormal HRV and HRT parameters with respect to normal subjects. Cardiac autonomic functions might be involved in FMF patients, even in patients without cardiac symptoms.
11) Canpolat, 2011	_	Seventy-seven FMF patients and 30 age/gender-comparable healthy controls were included. All patients were attack-free, and subjects with disease or drugs that are known to alter cardiac electrophysiology were excluded. Electrocardiographic data were obtained and analyzed.	Twelve FMF patients had amyloidosis. QT and QTc intervals were within the normal ranges and similar between FMF patients and healthy controls. QT dispersion, the peak-to-end interval of T wave (Tpe), Tpe/QT, and Tpe/QTc ratios were significantly higher in FMF patients than in healthy controls. Patients with amyloidosis had significantly higher QT dispersion, Tpe, Tpe/QT, and Tpe/QTc than their counterparts without FMF. Levels of proteinuria were moderately correlated with QT dispersion, Tpe, Tpe/QT, and Tpe/QTc.	FMF patients may have an increased risk for arrhythmias.
12) Karaman, 2017	_	Asymptomatic FMF patients (n=30) of Turkish ancestry living in Germany and age-matched healthy controls (n=37) were prospectively assessed using 12-lead ECG.	Patients and controls were comparable in gender and body mass index, and patients had higher erythrocyte sedimentation rate (ESR), C-reactive protein (CRP) and serum amyloid A (SAA) compared to controls (ESR: 23.7±14.3 vs. 16.1±13,3 mm/1sth, p=0.03, CRP: 0.73±0.9 vs. 0.26±0.4 g/dl, p=0.01, SAA: 3.14±4,8 vs. 0.37±0.3 mg/dl, p<0.01). No statistically significant difference between patients and controls, respectively, for Pdisp (43.7±11.9 vs. 47.1±11.2ms, p=0.23), QTdisp (65.9±12.3 vs. 67.6±12.7 ms, p=0.58) or corrected QTdisp (cQTdisp: 73.9±15.0 vs. 76.0±13.3 ms, p=0.55) was found. No correlation could be found between Pdisp or QTdisp or cQTdisp and any of the biochemical markers of inflammation.	FMF patients living in Germany show a Pdisp and QTdisp comparable to healthy controls, with no increased risk of atrial or ventricular arrhythmias indicated.
13) Giese, 2014	7.9 (4.9) "years"	This study included 66 patients with FMF and 58 healthy control subjects. Tp-Te and cTp-Te intervals and the cTp-Te/QT ratio were measured from the 12-lead electrocardiogram.	In electrocardiographic parameters, analysis of QT, QT dispersion, corrected QT (QTc), and QTc dispersion were similar between the groups. The Tp-Te and cTp-Te intervals and Tp-Te/QT and cTp-Te/QT ratios were significantly prolonged in FMF patients. Multivariate linear regression analyses indicated that erythrocyte sedimentation rate was an independent predictor of a prolonged cTp-Te interval.	The study revealed that when compared with control subjects, Tp-Te and cTp-Te intervals and cTp-Te/QT ratio were increased in FMF patients.
14) Ahbap, 2015	4.65 (3.19) "years"	The study included 69 patients with FMF and 71 healthy subjects as controls.	No statistically significant differences were found between the groups in QT dispersion, corrected QT dispersion, and systolic–diastolic function of the left ventricle parameters. During the 12 months of follow-up, no ventricular arrhythmias were documented in either group.	The study found that diastolic dysfunction was not associated with QT dispersion. No difference in QT markers was observed in cases and controls.

Outcomes

**Figure 2:**
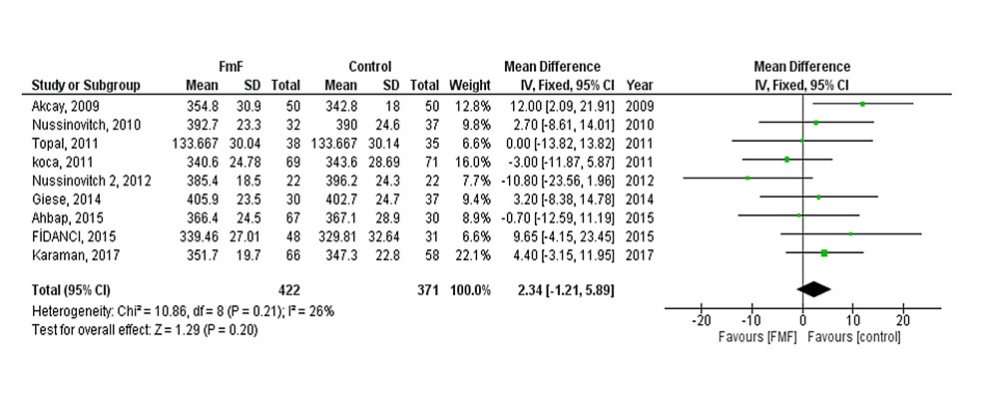
QT interval (QT) outcome

QT interval (QT): The pooled effect showed no statistically significant difference between the FMF and control groups (MD= 2.34, 95% CI = -1.21 to 5.89, p-value = 0.20). No heterogeneity was observed (P = 0.21, I² = 26%) (Figure 2). No publication bias was observed. We did subgroup analysis based on age (children and adults). No statistically significant difference between the FMF and control groups was found in both children and adults (MD 2.18, 95% CI = -10.01-14.37, p = 0.73) and (MD 2.82, 95% CI = -1.22-6.85, p = 0.17), respectively.

**Figure 3:**
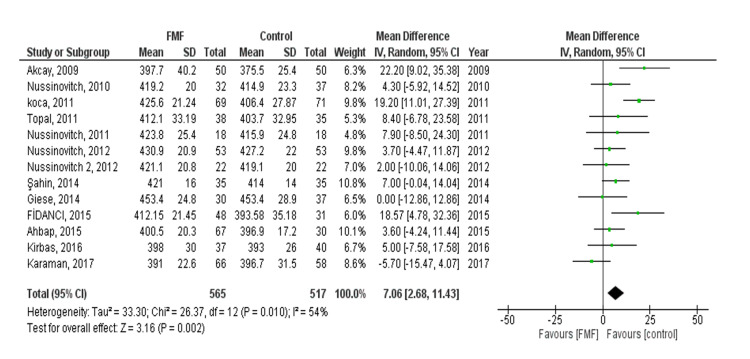
Corrected QT (QTc) outcome

QTc: The pooled effect showed a statistically significant difference between the FMF and control groups (MD= 7.06, 95% CI = 2.68 to 11.43, p-value = 0.002). We observed heterogeneity (P = 0.010, I² = 54%) (Figure 3). Heterogeneity was managed after doing leave one out test by eliminating (Koca, 2011, P = 0.12, I² = 33%), and the results showed statistically significant difference between FMF and control groups (MD= 5.51, [95% CI = 1.67 to 9.35], p-value= 0.005). There was no evidence of publication bias. Subgroup analysis was done based on age; a statistically significant difference between the FMF and control groups was found in both children and adults (MD 14.19, 95% CI = 5.25-23.12, p = 0.002) and (MD 3.96, 95% CI = 0.52-7.41, p = 0.02), respectively.

**Figure 4:**
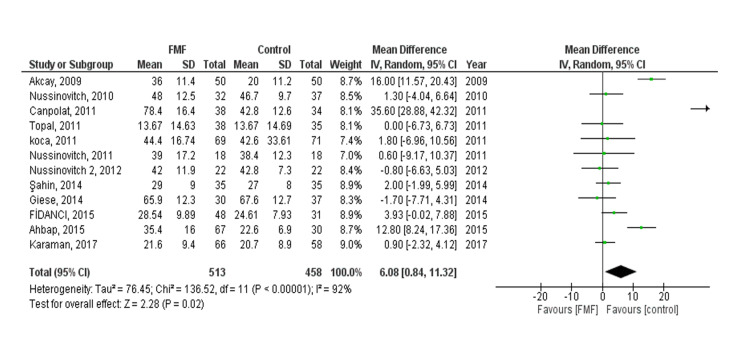
QT dispersion (QTd) outcome

QTd: The pooled effect showed a statistically significant difference between the FMF and control groups (MD 6.08, 95% CI = 0.84-11.32, p = 0.02). We observed heterogeneity (P < 0.00001, I² = 92%) that was not solved by leaving one out of a test or subgroup analysis (Figure 4). There was no evidence of publication bias. Subgroup analysis was done based on age, a statistically significant difference between the FMF and control groups was found in both children and adults (MD 2.86, 95% CI = 0.19-5.54, p = 0.04) and (MD 7.23, 95% CI = 0.08-14.37, p = 0.05), respectively.

**Figure 5:**
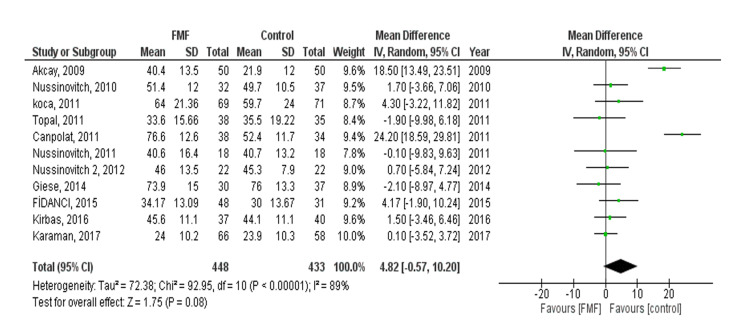
Corrected QT dispersion (QTcd)

QTcd: The pooled effect showed no statistically significant difference between the FMF and control groups (MD= 4.82, 95% CI = -0.57 to 10.20, p-value = 0.08). We observed heterogeneity (P < 0.00001, I² = 89%) that was not solved by leaving one out of the test or subgroup analysis (Figure 5). No publication bias was observed. After doing subgroup analysis based on age, no statistically significant difference between the FMF and control groups was found in both children and adults (MD 4.22, 95% CI = -0.50-8.95, p = 0.08) and (MD 4.91, 95% CI = -1.57-11.39, p = 0.14), respectively.

Discussion

FMF is a chronic disease that is characterized by recurrent attacks of inflammation that manifest with fever, abdominal pain, and arthritis [11]. Our analysis showed a statistically significant association between FMF and the prolongation of QTc and QTd intervals compared with the control group. Similarly, subgroup analysis for age showed a statistically significant association between FMF and prolongation of QTc and QTd intervals compared with the control group in both children and adults. Whereas QT interval and cQTd did not significantly differ between FMF and control groups.

These results were consistent with Akcay et al. regarding QTc prolongation and increased QTd [9]. This difference was attributed to the increased inflammatory activity and associated atherosclerosis that occurs with FMF. This was evidenced by the elevated levels of C-reactive protein (CRP) and erythrocyte sedimentation rate (ESR) among FMF patients compared to healthy controls. In addition, several studies have indeed demonstrated an association between the chronic inflammatory state of FMF and atherosclerosis [12,13], further accusing atherosclerosis of QTc interval prolongation and increased QTd. Koca et al. also showed a prolonged QTc interval. However, they did not find a correlation between inflammatory markers and electrocardiographic (ECG) parameters in FMF patients [14]. They attributed this to the shorter disease duration of their participants and the ECG measurements being taken during an attack-free period.

Amyloidosis also appears to contribute to increased QTd in FMF patients. Ahbap et al. found that QTd significantly increased in FMF patients, particularly those with amyloidosis, compared to FMF without healthy controls [15]. On the other hand, Nussinovitch et al. found no correlation between amyloidosis in FMF and increased QTd [16]. Of note, both studies had a low sample size for FMF patients with amyloidosis of 12 and 18, respectively, warranting further research.

Contradictory to our results, Topal et al. found no significant difference between FMF patients and healthy controls in all ventricular repolarization parameters [17]. Their findings may be due to the significant discrepancy in age between the FMF patients and healthy controls in their study. Similarly, Giese et al. also found no association between FMF and ECG parameters [18]. These variations between the different studies included in our analysis may be explained by the differences in age, duration of disease, colchicine therapy, or amyloidosis between studies.

Many studies have shown that prolonged QTc is associated with increased mortality, particularly among patients with chronic inflammatory diseases, such as rheumatoid arthritis [6,19,20]. One mechanism that underlies this association is the one described by Zabel et al. that prolonged QTc interval is associated with depolarizations during phases 2 and 3 of the action potential in animal models, thus impeding the process of repolarization [21]. Such early and incomplete action potentials can result in lethal ventricular arrhythmias such as torsade de pointes, ultimately resulting in ventricular fibrillation and sudden cardiac death [22,23]. QTc interval prolongation is also associated with increased mortality among patients with acute myocardial infarction [24]. Indeed, Gendelman et al. have demonstrated that FMF patients are at increased risk of mortality due to ischemic heart diseases, echoing the importance of QTc interval as a prognostic factor for FMF patients [25].

Although QTc interval and QTd were found to be significantly different between FMF patients and controls in our study, the results among the studies involved were inconsistent. This can be attributed to the different timing of ECG measurements during disease activity for FMF patients. Results can be influenced depending on whether the measurements were recorded during an exacerbation or attack-free period. In addition, different approaches to ECG measurements used between studies, either manual or computerized, can be a contributing factor. Furthermore, patient compliance with colchicine treatment can have a role in the in QTc interval and QTd variation. Colchicine reduces inflammatory markers in FMF patients and has a beneficial effect on cardiac arrhythmias [26]. However, one study demonstrated that, for FMF patients, being colchicine-sensitive or resistant has no significant effect on QT interval variability, warranting further research in that regard [8].

Future Implications

Regular follow-up and electrocardiographic and echocardiographic monitoring of FMF patients regarding their ventricular repolarization parameters is needed to allow for early detection and management of ventricular arrhythmias.

Strengths

The overall quality was high in most of the studies included in our analysis. The analysis included multiple studies, allowing for a fairly high combined sample size.

Limitations

Regarding quality assessment, two of the 14 included studies in the meta-analysis were of poor quality. The effect of colchicine on the outcomes of the patients was not investigated in the included studies. Although most of the included studies are case-control studies that included FMF patients and matched controls for age, sex, and comorbidities, other included studies did not give attention to matched controls for age, sex, and comorbidities, so prospective multicenter randomized studies with larger sample sizes and longer follow-up periods giving attention to matched controls for age, sex, and comorbidities are needed to further evaluate the relationship between FMF and ventricular repolarization parameters and to assess the effect of colchicine on the QT markers in FMF patients.

## Conclusions

Our analysis revealed a statistically significant link between FMF and prolonged QTc and QTd, but no statistically significant difference was observed in QT and QTcd. Therefore, FMF patients are at increased risk of developing arrhythmia. As a result, FMF patients should be scheduled for frequent follow-ups to avoid developing arrhythmias. More randomized multicenter trials are needed to confirm our findings and to establish the association between FMF and subsequent arrhythmia.
